# A Novel Erythrocyte Binding Protein of *Plasmodium vivax* Suggests an Alternate Invasion Pathway into Duffy-Positive Reticulocytes

**DOI:** 10.1128/mBio.01261-16

**Published:** 2016-08-23

**Authors:** Francis B. Ntumngia, Richard Thomson-Luque, Letícia de Menezes Torres, Karthigayan Gunalan, Luzia H. Carvalho, John H. Adams

**Affiliations:** a Center for Global Health and Infectious Diseases Research, College of Public Health, University of South Florida, Tampa, Florida, USA; b Centro de Pesquisas René Rachou/FIOCRUZ, Belo Horizonte, Minas Gerais, Brazil; c Laboratory of Malaria and Vector Research, National Institute of Allergy and Infectious Diseases, National Institutes of Health, Rockville, Maryland, USA

## Abstract

Erythrocyte invasion by malaria parasites is essential for blood-stage development and an important determinant of host range. In *Plasmodium vivax*, the interaction between the Duffy binding protein (DBP) and its cognate receptor, the Duffy antigen receptor for chemokines (DARC), on human erythrocytes is central to blood-stage infection. Contrary to this established pathway of invasion, there is growing evidence of *P. vivax* infections occurring in Duffy blood group-negative individuals, suggesting that the parasite might have gained an alternative pathway to infect this group of individuals. Supporting this concept, a second distinct erythrocyte binding protein (EBP2), representing a new member of the DBP family, was discovered in *P. vivax* and may be the ligand in an alternate invasion pathway. Our study characterizes this novel ligand and determines its potential role in reticulocyte invasion by *P. vivax* merozoites*.* EBP2 binds preferentially to young (CD71^high^) Duffy-positive (Fy^+^) reticulocytes and has minimal binding capacity for Duffy-negative reticulocytes. Importantly, EBP2 is antigenically distinct from DBP and cannot be functionally inhibited by anti-DBP antibodies. Consequently, our results do not support EBP2 as a ligand for invasion of Duffy-negative blood cells, but instead, EBP2 may represent a novel ligand for an alternate invasion pathway of Duffy-positive reticulocytes.

## IMPORTANCE

For decades, *P. vivax* infections in humans have been defined by a unique requirement for the interaction between the Duffy binding protein ligand of the parasite and the Duffy blood group antigen receptor (DARC). Recent reports of *P. vivax* infections in Duffy-negative individuals challenge this paradigm and suggest an alternate pathway of infection, potentially using the recently discovered EBP2. However, we demonstrate that EBP2 host cell specificity is more restricted than DBP binding and that EBP2 binds preferentially to Duffy-positive, young reticulocytes. This finding indicates that this DBP paralog does mediate a Duffy-independent pathway of infection.

## Observation

Malaria caused by *Plasmodium vivax* is the most predominant form of malaria outside Africa, with over 130 million clinical cases annually (1, 2). Unlike *Plasmodium falciparum* malaria, *P. vivax* blood-stage infection is limited to reticulocytes and individuals who are positive for the Duffy blood group antigen (Fy), also known as the Duffy antigen receptor for chemokines (DARC) (3, 4). Preference for this blood cell type is believed to be mediated by specific ligand-receptor interactions between the parasite merozoites and the host reticulocytes during the invasion process (5, 6). It is believed that the *P. vivax* Duffy binding protein (DBP) on the merozoite interacts with DARC on the reticulocyte surface, precipitating the junction formation step necessary for invasion. Historically, the vital need for the DBP-DARC interaction was evident from the virtual absence of *P. vivax* malaria in populations with a high prevalence of DARC negativity (3, 7). However, recent studies have reported evidence of Duffy (Fy)-independent invasion of human reticulocytes (8, 9). In Madagascar, with a mixture of Duffy-positive (Fy^+^) and -negative (Fy^−^) populations of diverse ethnic backgrounds, there was a significant reduction in the prevalence of clinical *P. vivax* malaria in Duffy-negative compared with Duffy-positive individuals (8). Similarly, in the Brazilian Amazon, two cases of clinical *P. vivax* malaria were observed in Duffy-negative samples obtained from Rondonia state (9). From these historically anomalous cases, it is not clear if they represent random isolated infections that have always occurred, if they are new phenomena related to *P. vivax* evolving at this time to use alternate DARC-independent pathways for invasion, or if DBP remains the critical invasion ligand using alternate receptors for invasion.

Recent studies have identified a *P. vivax* DBP homolog erythrocyte binding protein (termed here EBP2) that is the type of novel ligand anticipated in an alternate invasion pathway to DBP (10–12). EBP2 has the key conserved domain features characteristic of the EBP superfamily, including the region II or Duffy binding-like (DBL) ligand domain, considered essential for receptor recognition and merozoite invasion (5). Nonetheless, the *P. vivax* DBP region II (DBPII) has surprisingly stronger similarity to its paralogs in *Plasmodium knowlesi* (≈70%) than to the newly discovered *P. vivax* EBP2 (50%) (see Fig. S1 in the supplemental material). Therefore, the conserved features of EBP2 suggest that it has a role in invasion while its differences in the key receptor binding domain suggest that it can facilitate an alternate invasion pathway to the DBP ligand. To shed light on this important question of whether EBP2 is a Duffy-negative or DARC-independent ligand, we adapted standard *in vitro* functional assays for DBP to characterize EBP2 receptor specificity. The data presented here provide a better understanding of the biological role of EBP2 in the invasion process and indicate that EBP2 does not explain the observed transmission of *P. vivax* in some Duffy-negative individuals.

### Antigen production.

**FIG 1:**
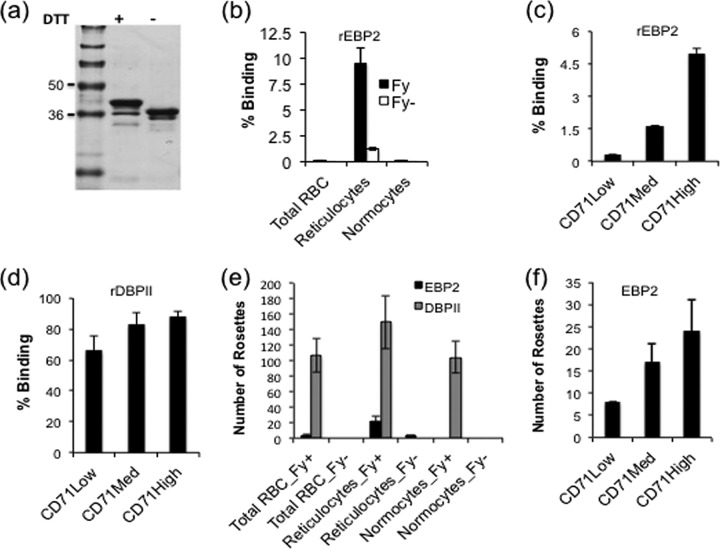
Production and erythrocyte binding properties of recombinant EBP2. (a) Coomassie blue-stained SDS-PAGE gel showing differential mobility of reduced (dithiothreitol-positive [DTT^+^]) and refolded (DTT^−^) recombinant EBP2. Mobility shift is a simple indicator of native conformation of the refolded antigen. (b to d) Recombinant EBP2 (b and c) and recombinant DBPII (d) binding to different red blood cell types and reticulocyte subpopulations, respectively, by flow cytometry. (e) COS7 cell surface-expressed EBP2 and DBPII binding to different Duffy-positive (Fy^+^) and Duffy-negative (Fy^−^) erythrocyte types. (f) COS7 cell surface-expressed EBP2 binding to different reticulocyte subpopulations. Bars show mean percentages of red blood cells with bound antigen and mean numbers of rosettes in 30 microscope fields at a magnification of ×20 in the flow cytometry and COS7 assays, respectively. Error bars represent ±standard deviations from two independent experiments. All experiments were performed with blood from at least two different donors. DBPII was used as a control antigen.

The gene sequence coding for the DBL domain of EBP2 (amino acids 159 to 485) (see Fig. S1 in the supplemental material) from GenBank (accession number KC987954) (10) was codon optimized for *Escherichia coli* expression, commercially synthesized, and cloned into an expression vector (pET21a^+^) with a C-terminal His tag to facilitate purification by affinity chromatography. Recombinant EBP2 was expressed in bacteria, purified from inclusion bodies, and refolded by rapid dilution as previously described (13, 14). The refolded antigen was evaluated for native conformation by separating refolded and denatured antigens by SDS-PAGE. The refolded antigen migrated at the expected size of 37 kDa compared to the denatured antigen at 39 kDa (Fig. 1a). This mobility shift on the gel is a simple indication of the formation of disulfide bonds in the refolded antigen.

### EBP2 binds to human reticulocytes.

To further analyze EBP2 for functional ligand activity, the refolded recombinant antigen was tested for binding to different erythrocyte types by a highly sensitive flow cytometry binding assay (15). First, reticulocytes were isolated from buffy packs (Interstate Blood Bank, Memphis) by immunomagnetic sorting using CD71 beads (Miltenyi Biotec). Briefly, blood cells (60 to 70 ml) were washed in McCoy’s 5A medium (Sigma) by centrifugation at 2,500 × *g* for 5 min. The washed cell pellet was filtered on a NEO1 leukocyte reduction filter (Haemonetics), mixed with an equal volume of cold autoMACS running buffer (Miltenyi Biotec), and incubated with anti-human CD71 microbeads (Miltenyi Biotec) for 30 min at 4°C. Enrichment for CD71-positive cells (reticulocytes) was performed on an autoMACS Pro Separator (Miltenyi Biotec) under positive-selection mode (Posselds), and purity was determined by new methylene blue staining of thin smears. The purity level of the reticulocytes was generally >90%. Subpopulations of the enriched reticulocytes (CD71^low^, CD71^med^, and CD71^high^) were isolated by staining with CD71-allophycocyanin (APC) (Miltenyi Biotec) and sorted on a BD FACSAria II cell sorter with a 70-µm nozzle (see Fig. S2 in the supplemental material). For the binding assay, 1 µl of total red blood cells, reticulocytes, or reticulocyte-depleted erythrocytes (normocytes) was incubated for 90 min with 5 µg/ml of refolded antigen in 100 µl of phosphate-buffered saline (PBS)–1% bovine serum albumin (BSA) at room temperature (RT) while shaking. Unbound antigen was washed off with PBS-1% BSA, and the cells were incubated with mouse anti-His Alexa Fluor 488-conjugated antibody (Qiagen) in the dark for 1 h at 4°C. After another washing step, the cells were suspended in wash buffer, and erythrocyte binding was quantified by analyzing 100,000 events on a BD Accuri C6 flow cytometer. Recombinant EBP2 bound to CD71^+^ reticulocytes with a preference for immature (CD71^high^) and Duffy-positive (Fy^+^) reticulocytes. Only minimal binding was observed with Fy^−^ reticulocytes, and no binding was observed with total red blood cells or to normocytes (Fig. 1b and c). Recombinant DBPII showed no significant differences in binding to young or mature reticulocytes (Fig. 1d). The binding specificity was confirmed by transient expression of EBP2 on the surface of COS7 cells as a recombinant transmembrane protein with enhanced green fluorescent protein (EGFP) fused to its cytoplasmic C terminus, as previously described (16, 17). Erythrocytes (reticulocytes or normocytes) were added to each well in a 24-well plate and incubated for 2 h. Nonadherent cells were washed off, and binding was quantified by counting the number of rosettes in 30 microscope fields at a magnification of ×20. The binding pattern was similar to that observed by flow cytometry, with the surface-expressed antigen binding preferentially to immature (CD71^high^) and Duffy-positive (Fy^+^) reticulocytes but not to normocytes. Similarly, only minimal binding was observed with Duffy-negative (Fy^−^) reticulocytes (Fig. 1e and f). As expected, DBPII, which was used as a control, bound to both Duffy-positive reticulocytes and normocytes (Fig. 1e).

### EBP2 is antigenically distinct from DBP and sensitive to antibody inhibition.

**FIG 2:**
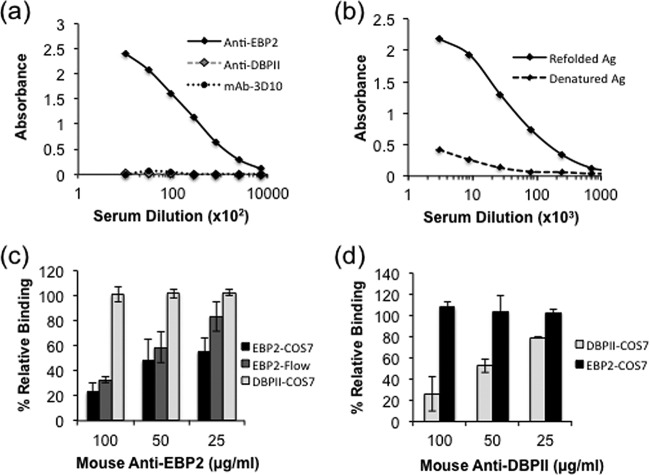
Immunogenicity and functional analysis of anti-EBP2 antibodies. (a and b) Antisera raised in mice (a) and rabbits (b) against recombinant EBP2 were tested by endpoint dilution for reactivity with the homologous antigen (Ag). Antigen preparations were allowed to adsorb onto wells of a microtiter plate and allowed to react with different dilutions of the antisera. Mouse anti-DBPII-Sal1 and mAb-3D10 were used as negative-control antibodies in the ELISA. Each point on the curves represents the mean OD from duplicate wells, while error bars represent ±standard deviations. (c and d) Mouse anti-EBP2 IgG (c) or anti-DBPII IgG (d) was tested against homologous and heterologous antigens in either the COS7 or flow cytometry binding assay for inhibition of EBP2 and DBPII reticulocyte binding. Each bar represents percent binding to reticulocytes in the presence of immune IgG relative to preimmune IgG. Error bars represent ±standard deviations.

Antiserum to refolded recombinant EBP2 prepared in mice and rabbits was used to characterize the antigenic properties of EBP2. Antibody titers were determined from immune sera by endpoint titer enzyme-linked immunosorbent assay (ELISA) as the reciprocal of the highest serum dilution giving an optical density (OD) twice that of the preimmune serum. Recombinant EBP2 was highly immunogenic in both animal species, with endpoint titers of 729,000 and 2 × 10^6^ in mice and rabbits, respectively (Fig. 2a and b). There was no reactivity of recombinant EBP2 with serum from DBPII-immunized mice and with a DBPII-specific monoclonal antibody, mAb-3D10 (14) (Fig. 2a). The rabbit polyclonal antibody showed very negligible reactivity with the denatured antigen compared with the refolded antigen, thus confirming the presence of conformational epitopes in the refolded antigen (Fig. 2b). To establish the functional activity of the anti-EBP2 antibodies, their potential to block EBP2-reticulocyte binding was evaluated. COS7 cell surface-expressed EBP2 or refolded recombinant EBP2 in solution was incubated with different concentrations of protein G-purified IgG from mouse serum raised against recombinant EBP2 prior to addition of human Duffy-positive reticulocytes. Binding inhibition was quantified by determining the number of rosettes (COS7 assay) or number of reticulocytes with bound EBP2 (flow cytometry) in the presence of IgG from immune serum relative to IgG from preimmune serum. A concentration-dependent inhibition of EBP2-reticulocyte binding was observed in both assays, but no inhibition of DBPII-reticulocyte binding was observed (Fig. 2c). Similarly, no inhibition of EBP2-reticulocyte binding was observed with mouse anti-DBPII IgG (Fig. 2d). These data confirm that the character and binding specificity of EBP2 are distinct from those of DBP.

### Conclusions.

Erythrocyte or reticulocyte invasion by malaria merozoites is central to blood-stage development of all malaria parasites. *Plasmodium vivax* has the distinctive characteristic of invading reticulocytes, with a particular preference for reticulocytes expressing the surface Duffy blood group antigen, and *P. vivax* malaria is rare in populations with high Duffy negativity (3, 4, 7). Not surprisingly, even short-term *ex vivo* culturing of *P. vivax* is difficult when the blood is not Duffy positive and enriched for reticulocytes (18, 19). However, an increasing number of studies in different countries in which malaria is endemic are revealing the common occurrence of *P. vivax* malaria in Duffy-negative individuals, which contradicts the historical paradigm (8, 9). This possibly suggests “discovery” of a preexisting phenomenon previously overlooked or possibly the emergence of an alternative Duffy-independent invasion pathway for *P. vivax* that may lead to an expansion of *P. vivax* malaria into Duffy-negative populations. Whatever the reason for the increasing frequency of these observations, it is a cause for concern.

Recent analysis of *P. vivax* genome from field isolates identified a second putative erythrocyte binding protein, EBP2, with characteristic features similar to those of DBP, which was absent in the initial Sal1 genome sequence (10–12, 20). The primary structure of EBP2 has all the characteristics typical of members of the EBP superfamily (5) but is phylogenetically distant from *P. vivax* DBP (10). Therefore, it is logical to suggest that EBP2 has a functional role distinct from that of DBP in receptor recognition and invasion of reticulocytes. However, a lack of genetic similarity of the genes for EBP2 and DBP suggests that EBP2 is not a recent duplication, its apparent low frequency of single nucleotide polymorphisms (SNPs) indicates that it is unlikely to be under the same level of immune selective pressures as DBP, and lack of conservation of its C-terminal cytoplasmic domain suggests a different internal linkage to the intracellular merozoite invasion motility machinery (10, 11).

In this study, we characterized the functional and antigenic properties of the DBL domain of EBP2 to help determine its potential to be an invasion ligand and mediate a Duffy-independent invasion pathway. Contrary to expectations, the cell binding specificity of EBP2 is more restricted than that of DBP, and accordingly, its functional properties lack those anticipated of a ligand to mediate an alternate, Duffy-independent invasion pathway. Specifically, EBP2 binds exclusively to reticulocytes, in contrast to DBP, which can bind Duffy-positive normocytes as well (Fig. 1). Interestingly, DBP, unlike EBP2, has no preferential binding to the different reticulocyte subpopulations. These combined dual cell binding preferences of EBP2 unexpectedly augment the functional properties of DBP and reticulocyte binding proteins (RBPs), which are the well-characterized ligands of *P. vivax* (21, 22), thereby restricting instead of expanding the *P. vivax* host cell range. Such a binding profile is consistent with the historically defined *P. vivax* invasion phenotype restricted to Duffy-positive reticulocytes, and certainly, the lack of a novel binding preference is in line with the ancient character of the EBP2 gene duplication.

The inability of EBP2 to bind Duffy-positive normocytes indicates that the preference for Duffy-positive reticulocytes is not directly dependent on binding to DARC, as our data also showed minimal binding of EBP2 to Duffy-negative reticulocytes. Similarly, a recent study also showed evidence of EBP2 binding to both Duffy-positive and Duffy-negative erythrocytes, though at low frequency (23). During receptor binding, the DBP ligand domain has been shown to form a homodimer with the N-terminal extracellular domain of DARC (24). The critical functional contact residues of the DBP-DARC interaction have been identified, and especially important is a binding pocket with strong affinity for a sulfotyrosine of the DARC receptor (25–27). Although EBP2 and DBP have highly conserved cysteine residues and many other residues (see Fig. S1 in the supplemental material), only about 15% of the residues that make up the DBP binding site are conserved. A key missing element of EBP2 is the lack of contact residues in one of the regions that make up the sulfotyrosine binding site on DBP (see Fig. S1), which would greatly diminish the affinity of a possible interaction of DARC with EBP2. In addition to the functional differences, DBP and EBP2 are clearly distinct antigenically, as highly inhibitory anti-DBP antibodies did not react with EBP2 and could not inhibit EBP2-reticulocyte binding. Conversely, anti-EBP2 antibodies effectively blocked EBP2-reticulocyte binding (Fig. 2c) but did not affect DBP-DARC interaction. Therefore, it is possible that EBP2 may function as an alternate ligand for *P. vivax* invasion of reticulocytes when the principal ligand, DBP, is blocked by immune antibody.

Our data also show a preference for binding of immature (CD71^high^) reticulocytes by EBP2. This reticulocyte subpopulation is generally restricted to the bone marrow (28) and was recently demonstrated to be the preferred cell type for invasion by *P. vivax* merozoites (29). Interestingly, *P. vivax* bone marrow infections have been reported in patients, even in cases with negative peripheral blood smears (30). Therefore, the observed host cell binding phenotype of EBP2 supports the growing evidence that *P. vivax* merozoite invasion of reticulocytes helps this parasite exploit a niche environment in the bone marrow.

In summary, we have characterized EBP2 as a functionally and antigenically distinct *P. vivax* ligand that binds exclusively to reticulocytes and has a strong preference for Duffy-positive reticulocytes. Based on these data, we propose that EBP2 and DBP are antigenically distinct but functionally redundant ligands that use different receptors while still retaining specificity for invasion of Duffy-positive reticulocytes. If EBP2 mediates an alternate invasion pathway, it seems more likely to be as a secondary pathway when immune inhibition blocks the principal DBP-DARC pathway. It remains to be determined whether the weak interaction of EBP2 with Duffy-negative reticulocytes observed in the *in vitro* functional assays is sufficient to facilitate invasion *in vivo*, although the lack of prevalent SNPs suggests a lack of significant immune exposure. The data presented here are an important step toward a better understanding of the biological role of EBP2 in the invasion process. Further research is necessary to determine the role of EBP2 in binding to Duffy-negative reticulocytes and its potential as a ligand mediating an alternate invasion pathway for *P. vivax* and to identify the receptor for this protein on reticulocytes.

## SUPPLEMENTAL MATERIAL

Figure S1  Alignment of amino acid sequence representing the DBL domains of *P. vivax* DBPII (PvDBPII) and PvEBP2. Shading, conserved cysteine residues; uppercase, identical residues; lowercase black, conserved amino acid substitutions; lowercase gray, nonconserved amino acid substitutions; underlining, DARC binding sites in DBPII; asterisk, major DBPII-DARC contact residues (adapted from the work of J. Hester et al., PLoS Negl Trop Dis **7:**e2569, 2013, http://dx.doi.org/10.1371/journal.pntd.0002569, and J. D. Batchelor et al., PLoS Pathog **10:**e1003869, 2014, http://dx.doi.org/10.1371/journal.ppat.1003869). Download Figure S1, TIF file, 0.7 MB

Figure S2  CD71^+^ buffy coat reticulocytes (1 × 10^9^), previously enriched with an autoMACS Pro Separator, were stained with CD71-APC (Miltenyi Biotec) and sorted into three subpopulations (P4-CD71^low^, P5-CD71^med^, and P6-CD71^high^) on a BD FACSAria II cell sorter. The gating was designed to avoid overlap of the distinct CD71 subpopulations. The flow rate for cell sorting was set at 10,000 events/s on a purity mode. Sorting efficiency was over 90%, and the purity of each subpopulation was 83% for P4-CD71^low^, 97% for P5-CD71^med^, and 95.7% for P6-CD71^high^. All sorting was performed in a precooled chamber at 4°C to avoid room-temperature maturation of the immature reticulocytes. Analysis was performed using BD FACSDiva v6 software. Download Figure S2, PDF file, 1.9 MB
