# Supplementary figures and images for: A Novel Erythrocyte Binding Protein of Plasmodium vivax Suggests an Alternate Invasion Pathway into Duffy-Positive Reticulocytes
Source: mBio. 2016 Aug 23;7(4):e01261-16. doi: 10.1128/mBio.01261-16 (PMC4999553; doi:10.1128/mBio.01261-16)

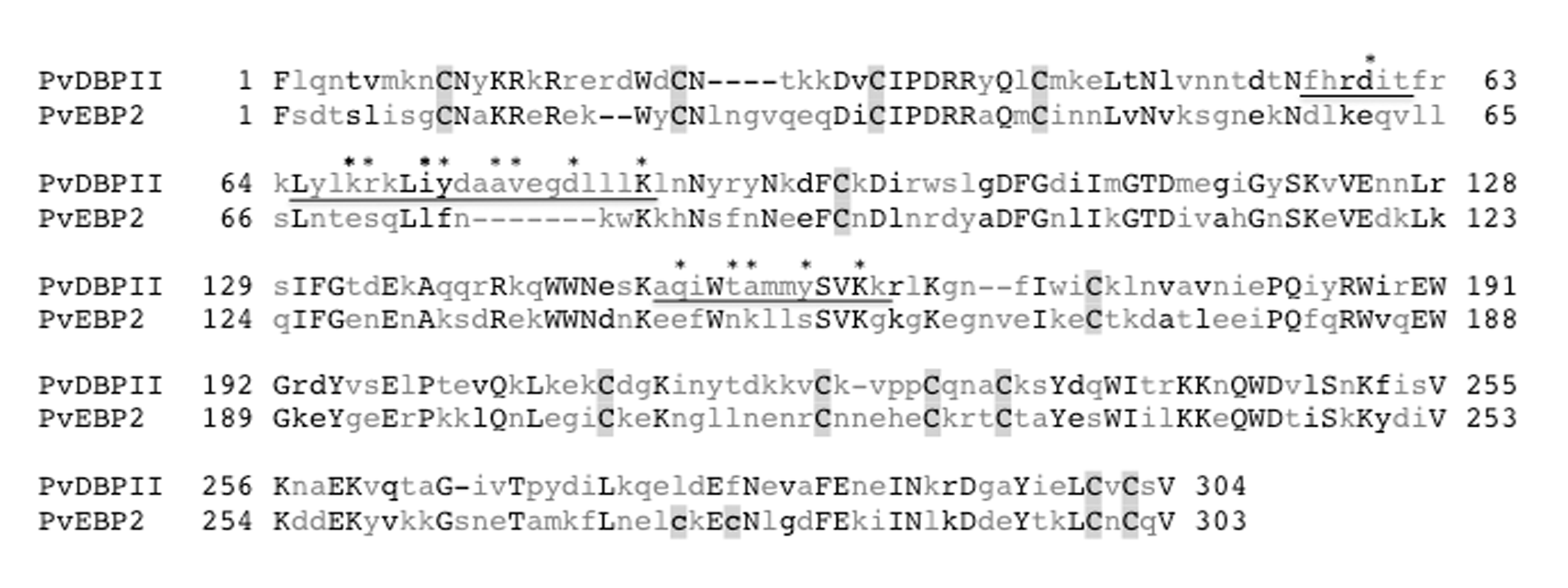

Supplement: Figure S1 — Alignment of amino acid sequence representing the DBL domains of P. vivax DBPII (PvDBPII) and PvEBP2. Shading, conserved cysteine residues; uppercase, identical residues; lowercase black, conserved amino acid substitutions; lowercase gray, nonconserved amino acid substitutions; underlining, DARC binding sites in DBPII; asterisk, major DBPII-DARC contact residues (adapted from the work of J. Hester et al., PLoS Negl Trop Dis 7:e2569, 2013, http://dx.doi.org/10.1371/journal.pntd.0002569, and J. D. Batchelor et al., PLoS Pathog 10:e1003869, 2014, http://dx.doi.org/10.1371/journal.ppat.1003869). Download [file mbo004162958sf1.tif]

Presort

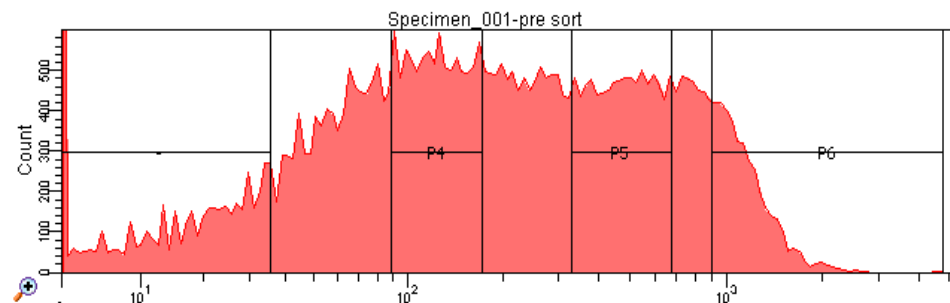

CD71<sup>Low</sup>

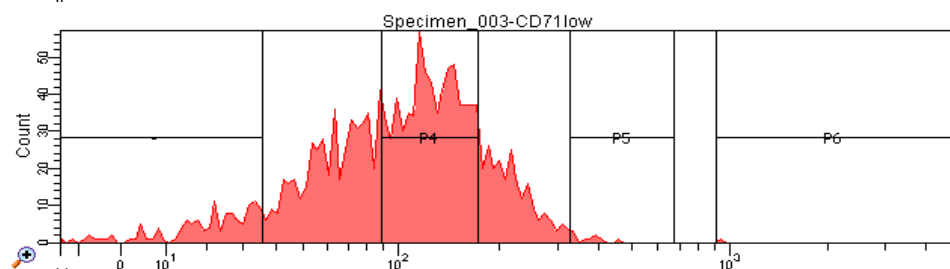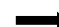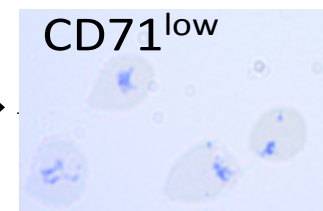

CD71<sup>Med</sup>

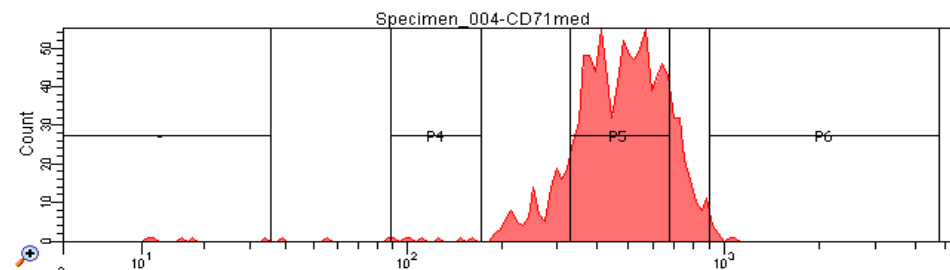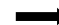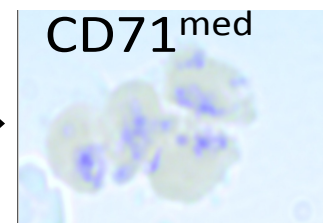

CD71<sup>High</sup>

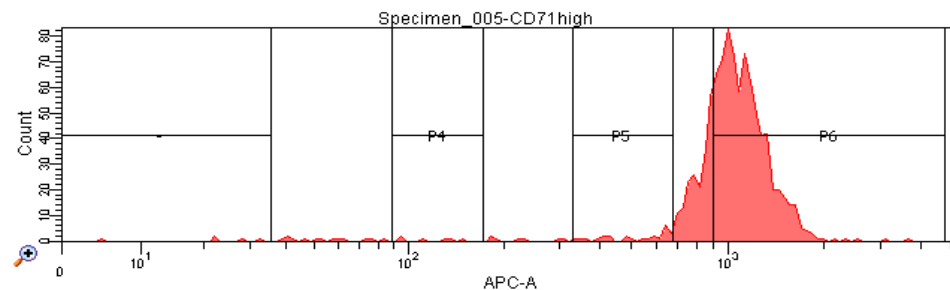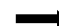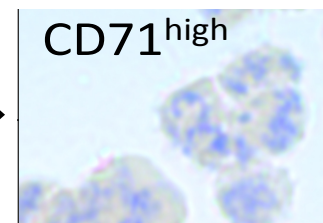

Supplement: Figure S2 — CD71+ buffy coat reticulocytes (1 × 10^9^), previously enriched with an autoMACS Pro Separator, were stained with CD71-APC (Miltenyi Biotec) and sorted into three subpopulations (P4-CD71low, P5-CD71med, and P6-CD71high) on a BD FACSAria II cell sorter. The gating was designed to avoid overlap of the distinct CD71 subpopulations. The flow rate for cell sorting was set at 10,000 events/s on a purity mode. Sorting efficiency was over 90%, and the purity of each subpopulation was 83% for P4-CD71low, 97% for P5-CD71med, and 95.7% for P6-CD71high. All sorting was performed in a precooled chamber at 4°C to avoid room-temperature maturation of the immature reticulocytes. Analysis was performed using BD FACSDiva v6 software. Download [file mbo004162958sf2.pdf]
